# Clinical audit: long-term follow-up of women with genital lichen sclerosus

**DOI:** 10.1186/1753-6561-6-S4-P45

**Published:** 2012-07-09

**Authors:** WC Ngu, C Green

**Affiliations:** 1 Ninewells Hospital & Medical School, University of Dundee, UK; 2 Ninewells Hospital & Medical School, UK

## Introduction

Genital lichen sclerosus (LS) is a chronic inflammatory disease usually managed with intermittent potent topical corticosteroids.5% of women with untreated vulval LS go on to develop squamous cell carcinoma (SCC).

Current guidelines from the British Association of Dermatologists (BAD) state that patients on topical corticosteroids should be reviewed at least annually by their General Practitioner (GP) [1]. Previous work has shown that many women discharged from secondary to primary care are lost to follow-up [2].

## Methoda

An anonymised questionnaire was sent to sixty women with genital LS discharged from our regional vulval clinic more than a year previously. Caldicott Guardian approval was obtained for the study.

## Results

Forty-five patients (75%) returned the questionnaire. Seventy one percent of patients had not attended their GP for follow-up. Of the 17 patients who had seen their GP, only 7 had had their vulval area examined. Only 53% of patients self-examined their vulval area. 48% were not aware of the need to report any area of persistent abnormality to their GP. 28 patients were still using topical corticosteroid - 24 of these potent or ultrapotent steroid. 48.9% of all respondents thought a 30g tube of strong steroid should last a year, 26.7% three months, 13.3% one month and 11.1% were unsure. Only 30 patients (66.7%) were aware of the increased risk of skin cancer associated with vulval LS, the remainder being unaware or uncertain.

## Conclusions

Our study has highlighted worrying deficiencies in the long-term follow-up of this patient group. We have devised a brief patient information leaflet to be provided to both the patient and their GP at discharge to detail the small increased cancer risk, the need for monthly self-examination, clear instructions on the use of topical corticosteroids and the need for annual review by their GP. Hopefully, this will lead to an increased quality of patient care. We then plan to repeat the audit after an appropriate time interval.
